# Alkylammonium Halides for Phase Regulation and Luminescence Modulation of Cesium Copper Iodide Nanocrystals for Light-Emitting Diodes

**DOI:** 10.3390/molecules29051162

**Published:** 2024-03-05

**Authors:** Wen Meng, Chuying Wang, Guangyong Xu, Guigen Luo, Zhengtao Deng

**Affiliations:** State Key Laboratory of Analytical Chemistry for Life Science, National Laboratory of Microstructures, College of Engineering and Applied Sciences, Nanjing University, Nanjing 210023, China; 17351930477@163.com (W.M.); wangchuying@yeah.net (C.W.); xuguangyong@smail.nju.edu.cn (G.X.); 502022340061@smail.nju.edu.cn (G.L.)

**Keywords:** copper halides, photoluminescence quantum yield, stability, surface passivation, white LEDs

## Abstract

All-inorganic cesium copper halide nanocrystals have attracted extensive attention due to their cost-effectiveness, low toxicity, and rich luminescence properties. However, controlling the synthesis of these nanocrystals to achieve a precise composition and high luminous efficiency remains a challenge that limits their future application. Herein, we report the effect of oleylammonium iodide on the synthesis of copper halide nanocrystals to control the composition and phase and modulate their photoluminescence (PL) quantum yields (QYs). For CsCu_2_I_3_, the PL peak is centered at 560 nm with a PLQY of 47.3%, while the PL peak of Cs_3_Cu_2_I_5_ is located at 440 nm with an unprecedently high PLQY of 95.3%. Furthermore, the intermediate-state CsCu_2_I_3_/Cs_3_Cu_2_I_5_ heterostructure shows white light emission with a PLQY of 66.4%, chromaticity coordinates of (0.3176, 0.3306), a high color rendering index (CRI) of 90, and a correlated color temperature (CCT) of 6234 K, indicating that it is promising for single-component white-light-emitting applications. The nanocrystals reported in this study have excellent luminescence properties, low toxicity, and superior stability, so they are more suitable for future light-emitting applications.

## 1. Introduction

In recent decades, metal halide perovskites (MHPs), which are a new generation of semiconductor materials, have shown enormous potential in numerous optoelectronic applications [1,2,3]. Halide perovskites have a general chemical formula of ABX_3_, where A = Cs^+^, methylammonium, or formammidinium; B = Pb^2+^, Sn^2+^, or Mn^2+^; and X = I^−^, Br^−^, or Cl^−^ [4,5]. Owing to their low-cost solution processing [6], broad substrate compatibility [7,8], wide wavelength tunability [9,10], low defect density [11,12,13], large absorption coefficient [14], and high photoluminescence quantum yields [15,16,17], these materials have been applied in many fields, such as in light-emitting diodes (LEDs) [18,19], displays [20], image sensors [21,22], lasers [23], field-effect transistors (FET) [24], solar cells [25,26], photodetectors [27,28], and photocatalysts [29]. Unique properties and impressive achievements have been demonstrated over the past several years. In particular, remarkable power conversion efficiencies of over 25% have been reported, surpassing commercialized cadmium telluride, polysilicon, and copper indium gallium selenide photovoltaic devices [30,31,32]. Unfortunately, the toxicity of lead is a major problem in CsPbX_3_-based optoelectronic devices regarding their widespread and scalable commercialization. To tackle the significant challenge of toxicity, an attractive research direction is the search for lead-free perovskite [33,34,35] and double-perovskite materials [36,37] to achieve environmentally friendly MHPs. Through theoretical calculations, the same group IV cations of Sn^2+^ and Ge^2+^ have been employed as replacements for Pb^2+^, firstly, due to their comparable ionic radii and identical valence. However, the easy oxidation of Sn and Ge from the +2 state to the +4 state makes these substitutions less promising for their industrialization for efficient, stable, and long-operation devices [38,39,40]. Bi^3+^ and Sb^3+^ have been considered to fabricate A_3_M_2_X_9_-configuration MHPs, which are equipped with a similar electronic configuration [41,42,43] and exhibit impressive characteristics. However, the lower PLQYs, long-term instability, poor optical properties, and complex technological process limit their potential application in modern industrialization. From the previous literature reported, the performance of PCEs for Sn-based perovskite solar cells is usually less than 10% [44], which is far below that of their lead-based perovskite counterparts. 

Although some lead-free MHPs have been researched, there is still a need for the development of stable and eco-friendly non-lead materials. Because of the cheap, economical, abundant, stable, and nontoxic features of the metal element copper, copper-based perovskite materials have attracted a great deal of attention, and many efforts have been devoted to the development of multifarious compounds. Therefore, a series of cesium copper halides with the chemical formulas Cs_α_Cu_β_X_α+β_ for Cu^+^ and Cs_α_Cu_β_X_α+2β_ for Cu^2+^, with excellent emission performance and remarkable optoelectronic properties, have been developed by using component engineering and various synthesis technologies. Very recently, all-inorganic Cs_2_CuX_4_ (X = Cl^−^, Br^−^, and I^−^) QDs were first prepared using the LARP technique at room temperature. Blue–green light with a high quantum yield and excellent stability was achieved, and it is worth pointing out that the molar ratio of the raw materials had a key role in the particle size and photoluminescence wavelength [45]. Apart from Cu^2+^-based materials, the recently developed low-dimensional all-inorganic Cu(I)-based crystals are very attractive, as they have stability in the air and are self-absorption-free due to a large Stokes shift. A battery made of Cs_3_Cu_2_I_5_ [46], CsCu_2_I_3_ [47], Cs_3_Cu_2_Cl_5_ [48], Cs_3_Cu_2_Br_5_ [49], Rb_2_CuBr_3_ [50], Rb_2_CuCl_3_ [51], K_2_CuCl_3_, and several organic–inorganic alternatives [52] has been fabricated using hot injection, antisolvent recrystallization, and solvent-assisted grinding processes [53]. Due to the similar composition of the Cs, Cu, and I elements and their soft crystal structures, chemical transformation between Cs_3_Cu_2_I_5_ and CsCu_2_I_3_ is an interesting conversion pathway among inorganic crystal structures, and some research groups have already achieved phase transformation. In 2019, Jun et al. investigated the 1D structure of CsCu_2_I_3_ and realized the dimensionality control of the Cs-Cu-I system, but only a PLQY of 8% centered at 560 nm was demonstrated [54]. Zhang et al. controlled the blue emitter (440 nm) of Cs_3_Cu_2_I_5_ and the yellow emitter (552 nm) of CsCu_2_I_3_ using a one-step route in ethanol at room temperature. As reported in the work by Feng et al., Cs_3_Cu_2_I_5_ polycrystal powders were first synthesized using a simple ball-milling method, and reversible PL emission was achieved upon exposure to/removal of water [55]. Because both 1D CsCu_2_I_3_ and 0D Cs_3_Cu_2_I_5_ are I-based phases, the possibility of mixing these two compounds is especially attractive for a wide range of applications. For example, Cui et al. demonstrated that ternary Cs_3_Cu_2_I_5_ nanocrystals and CsCu_2_I_3_ microrods could be synthesized via the hot-injection approach, and the emission change from blue to yellow indicated that Cs-Cu-I may have potential application in rapid anticounterfeiting [56]. These are different from the well-reported lead-based halides, which often undergo a typical halide exchange or migration reaction when mixed with different halide compositions of nanocrystals [57]. However, size and shape control have not been thoroughly studied for copper halide compounds. Additionally, few works have reported on the relationships among the luminescence tuning, structural evolution, and phase transition of Cs-Cu-I on the nanoscale. Considering that copper-based metal halides are promising in the area of light-emitting materials [58,59], the study of the structural rearrangement, morphology, tunable PL characteristics, and dimensionality of the Cs-Cu-I system could help to achieve efficient emission, large Stokes shifts, and admirable stability [60]. 

In this study, lead-free CsCu_2_I_3_ microrods (MRs) with yellow emission and Cs_3_Cu_2_I_5_ nanocrystals (NCs) with blue emission were prepared via a modified hot-injection strategy. By adding different amounts of OLA-I as a precursor, the phase and morphology were effectively adjusted by changing the feeding quantity. When no additional OLA-I was given, rod-like phase-pure CsCu_2_I_3_ MRs were more likely to be obtained, which provided broadband yellow emission (~580 nm) and a large Stokes shift with a high PLQY of up to 47.3%, owing to self-trapped exciton formation. Increasing the amount of OLA-I (2.2 M) promoted the formation of Cs_3_Cu_2_I_5_ with blue photoluminescence (~440 nm) and achieved the highest PLQY of 95.3%. Structural evolution occurred in the presence of a high dosage of oleylammonium iodine at the same reaction temperature, and a pure phase of Cs_3_Cu_2_I_5_ NCs was steadily obtained with a given quantity of 5 mmol. Importantly, this work provides a perspective on the growth and structural evolution from 1D CsCu_2_I_3_ MRs to 0D Cs_3_Cu_2_I_5_ NCs in a hot-injection reaction system. Additionally, we investigated the colors of their photoluminescence (PL) emission, which was helpful in adjusting their photoluminescence in LEDs. Specifically, a nearly white photoluminescence emission CsCu_2_I_3_/Cs_3_Cu_2_I_5_ composite could be realized with the appropriate amount of OLA-I, which provides a simple strategy for the potential WLED application of lead-free materials. The synthesis technology in this work can be extended to other copper-based ternary halides of Cs_3_Cu_2_Cl_5_ or Cs_3_Cu_2_Br_5_, thus significantly enhancing the development of high-performance, air-stable, nontoxic, and earth-abundant copper-based halides. 

## 2. Results and Discussion 

**Figure 1:**
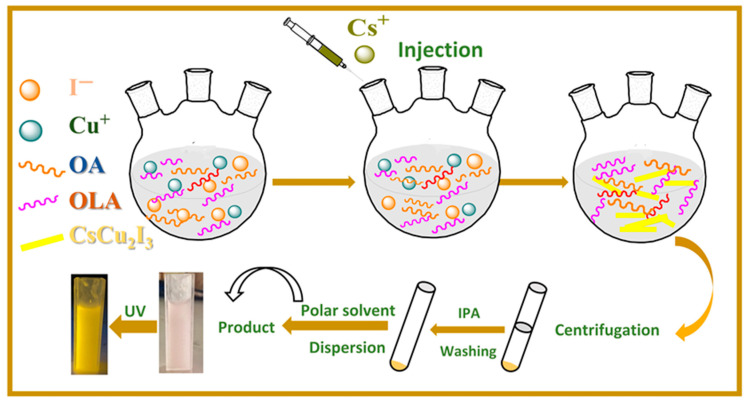
Schematic illustration of the synthetic process for cesium copper iodide with different OLA-I stoichiometric ratios through hot-injection technique.

By controlling the added amount of OLA-I, the phase of cesium copper iodide can be controlled to CsCu_2_I_3_ or Cs_3_Cu_2_I_5_. Through the classical hot-injection method, as illustrated in Figure 1, different amounts of oleylammonium iodide (OLA-I) were added into a precursor containing 1-octadecene (ODE), copper iodide, oleic acid (OA), and oleylammonium (OLA). The OLA-I precursor provided additional iodine ions to promote Cu^+^’s combination with Cs^+^ and I^−^ to form Cs_3_Cu_2_I_5_ NCs. Furthermore, it contributed to CsCu_2_I_3_’s conversion to Cs_3_Cu_2_I_5_ with the chemical reaction CsCu_2_I_3_ + 2CsI → Cs_3_Cu_2_I_5_, which was simultaneously stabilized using OLA and OA ligands. 

**Figure 2:**
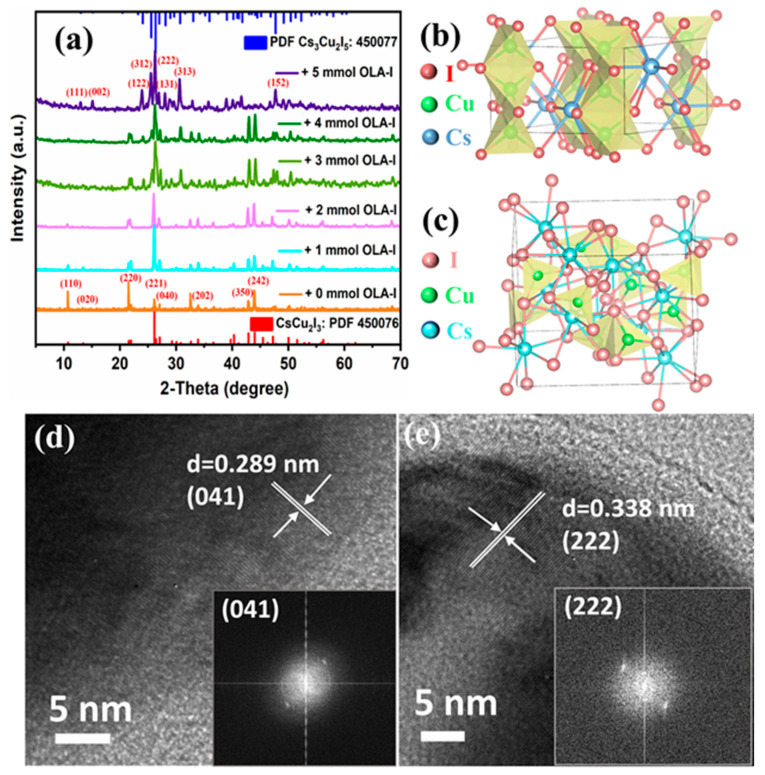
(**a**) XRD patterns of CsCu_2_I_3_ microscale crystals, CsCu_2_I_3_/Cs_3_Cu_2_I_5_ composites, and nanocrystalline Cs_3_Cu_2_I_5_. (**b**,**c**) Crystalline structures of 1D CsCu_2_I_3_ (face-sharing and edge-sharing Cu^+^I_4_ tetrahedra resulting in infinite double chains) and 0D Cs_3_Cu_2_I_5_ (containing Cu^+^I_4_ tetrahedra and Cu^+^I_3_ triangles). High-resolution transmission electron microscope (HR-TEM) images and corresponding FFT images of (**d**) CsCu_2_I_3_ MRs (regulated with 1 mmol OLA-I added) and (**e**) Cs_3_Cu_2_I_5_ NCs (regulated with 5 mmol OLA-I added).

X-ray diffraction (XRD) patterns were detected to confirm the structures of CsCu_2_I_3_ and Cs_3_Cu_2_I_5_, as shown in Figure 2a. As expected, in the XRD reflections for samples with 0, 1, and 2 mmol OLA-I added, the diffraction pattern matched well with the standard data of CsCu_2_I_3_ (JCPDS, No. 77-0069). This result indicated that the as-synthesized MRs could be predominantly crystallized in an orthorhombic structure with the Cmcm space group [61]. By careful examination, a series of strong diffraction peaks corresponding to the (110), (020), (220), (221), (040), (202), (350), and (242) crystal planes of the orthogonal-phase CsCu_2_I_3_ were found. Additionally, a new diffraction peak at an angle of 26.43°, corresponding to the (222) lattice plane of Cs_3_Cu_2_I_5_, could be seen clearly with more OLA-I added, indicating that the sample formed a mixture of CsCu_2_I_3_ and Cs_3_Cu_2_I_5_ (as shown in Figure S2). The new diffraction peaks of the samples corresponded well to the standard diffractions of Cs_3_Cu_2_I_5_ (JCPDS, No. 45-0077). The dominant diffraction peaks observed at 13.12°, 15.26°, 24.16°, 25.65°, 26.43°, 28.22°, 30.81°, and 47.83° could be assigned to the (111), (002), (122), (312), (222), (131), (313), and (152) lattice planes of orthorhombic Cs_3_Cu_2_I_5_, which crystallized in the Pnma space group [62,63]. Pure Cs_3_Cu_2_I_5_ NCs were collected with 5 mmol OLA-I, without other impurities, such as CsI and CuI, which indicated the satisfactory phase purity of the two components in the Cs-Cu-I system. In addition, low- and high-resolution transmission electronic microscopy (TEM) was used to characterize CsCu_2_I_3_ and Cs_3_Cu_2_I_5_ in different regions and the corresponding fast Fourier-transform (FFT) patterns are shown in Figure 2d,e. Moreover, as shown in Figure S3, the obtained low-magnification TEM images and high-magnification TEM images of another region in CsCu_2_I_3_ and Cs_3_Cu_2_I_5_ proved the integrity of the samples during the testing process. It can be seen that the samples and lattice fringes held up well under the attack of high-energy particle beams. The calculated spacing of the lattice fringes in different regions was consistent, which further demonstrated that the measured samples maintained their integrity under the impact of high-energy ion beams. The OLA-I-stimulated phase transition was attributed to the ionic nature of CsCu_2_I_3_ and the high solubility of I^−^ when exposed to the solvent. The eventual equilibrium phase between CsCu_2_I_3_ and Cs_3_Cu_2_I_5_ was determined by the dissolution and recrystallization of I^−^ in the reaction system. When more OLA-I was added into the reaction precursor, the phase transition was achieved when I^−^ was inserted into the CsCu_2_I_3_ crystalline framework. Thus, we conclude that the transformation process under this condition can be written as CsCu_2_I_3_ + 2CsI → Cs_3_Cu_2_I_5_. Both samples showed excellent crystallinity and explicit lattice fringe spacing. Specifically, the distance of the lattice fringe was measured to be 0.289 nm, corresponding to the (041) crystal face of CsCu_2_I_3_, and an interplanar distance of 0.338 nm was found for the (222) plane of the Cs_3_Cu_2_I_5_ orthorhombic phase. In order to better understand the coordination and dimensional structure of the Cs-Cu-I system, we examined the crystalline structures of 1D CsCu_2_I_3_ and 0D Cs_3_Cu_2_I_5_, as illustrated in Figure 2b,c. In the ribbon-like crystal structure for CsCu_2_I_3_, the Cu^+^ ions reside in the tetrahedrally coordinated center and Cu^+^I_4_ tetrahedrons share two sides with neighboring Cu^+^I_4_ tetrahedrons. The edge-sharing [Cu_2_I_3_]^−^ anionic ribbon is spatially surrounded and isolated by Cs^+^ atoms, forming a chain 1D structure. This unique localized structure contributes to the formation of self-trapping excitons. Moreover, the crystal structure of 0D Cs_3_Cu_2_I_5_ displays units of [Cu_2_I_5_]^3−^ composed of two types of Cu^+^ sites, a Cu^+^I_4_ tetrahedral site and a Cu^+^I_3_ trigonal site with one side shared, and each of the [Cu_2_I_5_]^3−^ units is separated by large Cs^+^ cations.

**Figure 3:**
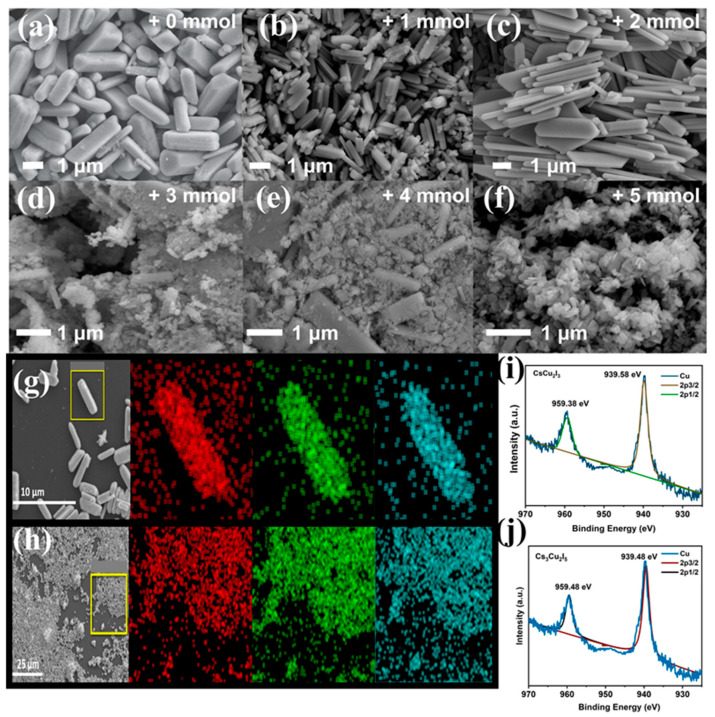
(**a**–**c**) SEM images of CsCu_2_I_3_ microscale crystals with OLA-I amounts of 0, 1, and 2 mmol. (**d**,**e**) SEM images of CsCu_2_I_3_/Cs_3_Cu_2_I_5_ composites with OLA-I amounts of 3 and 4 mmol. (**f**) SEM images of Cs_3_Cu_2_I_5_ nanocrystalline with OLA-I amount of up to 5 mmol. (**g**) Chemical composition revealed by EDS elemental mapping for CsCu_2_I_3_ MRs and (**h**) Cs_3_Cu_2_I_5_ NCs (red: Cs; green: Cu; blue: I). (**i**) High-resolution XPS spectrum of Cu (2p_1/2_ and 2p_3/2_) of CsCu_2_I_3_. (**j**) High-resolution XPS spectrum of Cu (2p_1/2_ and 2p_3/2_) of Cs_3_Cu_2_I_5_.

The morphology of the CsCu_2_I_3_ sample without additional OLA-I was characterized by SEM, as shown in Figure 3a, and elongated and homogeneous microrods with a length of ∼10 μm and a width of ∼1 μm were clearly depicted. Accompanied by additional OLA-I, smaller microrods were obtained, as shown in Figure 3b,c. The measured length and diameter distribution statistics of CsCu_2_I_3_ MRs controlled through the addition of 1 mmol OLA-I are shown in Figure S4a,b. The length ranged from roughly 0.8 μm to 1.7 μm, while the diameter was approximately aggregated around 0.34 μm. The results confirmed that the OLA-I was beneficial to promote the rapid crystallization process and control the size of the MRs, which was attributed to the strong Ostwald ripening effect [64]. The amount of I^−^ has an important influence in controlling the thermodynamic equilibrium of the reaction for CsCu_2_I_3_, and the participation of I^−^ can adjust the chemical potential in the reaction system [65]. Briefly, only smaller CsCu_2_I_3_ MRs were synthesized when the iodine element was abundant, despite the same quantity of prescribed Cs^+^ and Cu^+^ ions during the reaction process. When the addition amount was increased to more than 2 mmol, smaller particles of Cs_3_Cu_2_I_5_ NCs appeared, which aggregated around the CsCu_2_I_3_ MRs and exhibited an average nanoscale diameter of 40–80 nm. In Figure 3d,e, it can be seen that the CsCu_2_I_3_/Cs_3_Cu_2_I_5_ composites coexisted in the same system, and cesium copper iodide was crystallized to obtain a versatile composition by controlling the raw material of iodide ions. A high dosage of OLA-I was beneficial to produce abundant nanoparticles of Cs_3_Cu_2_I_5_ NCs, and when the quantity was increased to 5 mmol, only its phase was realized and the CsCu_2_I_3_ MRs disappeared, as shown in Figure 3f. The average size distribution statistics of Cs_3_Cu_2_I_5_ NCs were investigated, as shown in Figure S4c, which were consistent with the SEM results. 

To further characterize the element components and distribution of samples, energy-dispersive spectroscopy (EDS) mapping was carried out, as presented in Figure 3g,h. The uniform distribution of Cs, Cu, and I in the microrods and nanocrystals confirmed that the atoms were evenly distributed. Elemental analyses by EDS revealed that the ratios of Cs:Cu:I was 1:1.78:3.04 and 1:0.69:1.76, respectively, for samples synthesized with 0 and 5 mmol OLA-I added, which were attributed to CsCu_2_I_3_ and Cs_3_Cu_2_I_5_, respectively. Furthermore, the ratios of Cs:Cu:I for other samples with OLA-I are provided in Figure S1 (Supplementary Materials). From the spectra and calculation results, it can be deduced that the phase transition process from CsCu_2_I_3_ MRs to CsCu_2_I_3_/Cs_3_Cu_2_I_5_, and eventually presented as Cs_3_Cu_2_I_5_ NCs, can be regulated by adding different molar amounts of OLA-I (2.2 M) in the precursor solution. As the injection ratio increases, the adhesion of ligands on the surfaces of CsCu_2_I_3_ MRs can be reduced and this contributes to NCs’ growth. Additionally, with the addition of 2 and 3 mmol OLA-I, both 1D CsCu_2_I_3_ and 0D Cs_3_Cu_2_I_5_ could coexist in one system with a complete structural state, which was determined by the arrangement and rapid self-assembly behavior of the Cs-Cu-I MRs and NCs.

To determine the chemical components and the valence state of Cu in the two phases of the Cs-Cu-I system, X-ray photoelectron spectroscopy (XPS) measurement was conducted. Figure S5a presents the survey XPS spectrum of CsCu_2_I_3_ obtained by adding 1 mmol OLA-I. Obviously, the peaks are in good agreement, with Cs 3d, Cu 2p, and I 3d orbitals, and no other peaks for impurities are found. Furthermore, the Cu 2p_3/2_ peak consists of the main peak at 939.58 eV and another peak of Cu 2p_1/2_ located at 959.38 eV, which are attributed to the Cu^+^ state rather than the Cu^2+^ state (shown in Figure 3i), whose characteristic satellite peak is approximately situated at 943 eV. As seen from Figure S5b,d, the binding energies of Cs 3d_3/2_, 3d_5/2_, I 3d_3/2_, and I 3d_5/2_ were determined to be around 746.38 eV, 732.48 eV, 639.18 eV, and 627.68 eV, respectively. For Cs_3_Cu_2_I_5_, two main peaks appeared at 939.48 eV and 959.48 eV, which corresponded to Cu 2p_3/2_ and Cu 2p_1/2_ and were consistent with CsCu_2_I_3_. These results exclude the presence of divalent copper in both CsCu_2_I_3_ and Cs_3_Cu_2_I_5_ and demonstrate the advantageous reaction environment for the coordination structure. For both CsCu_2_I_3_ and Cs_3_Cu_2_I_5_, the Cs 3d, Cu 2p, and I 3d orbitals had similar shifts, which could be attributed to the 0D or 1D environment, leading to a different local chemical structure. 

**Figure 4:**
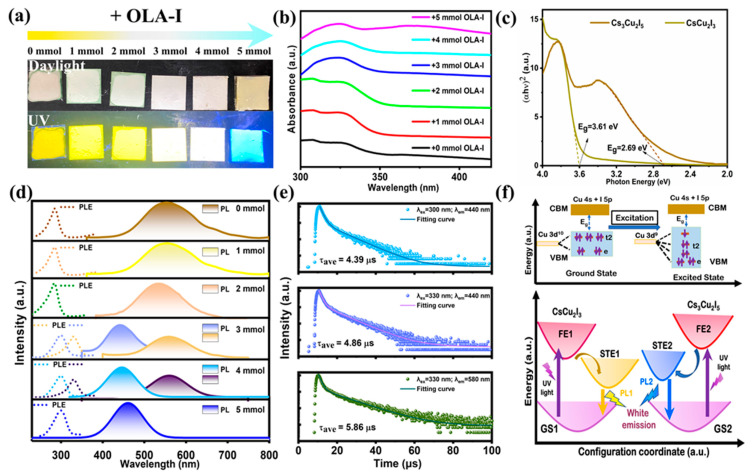
(**a**) Photographs of synthesized OLA-I treatment samples after dropping onto glasses (the upper image corresponds to daylight and the following corresponds to 300 nm UV irradiation). (**b**) UV–vis absorbance spectra of the synthesized CsCu_2_I_3_, CsCu_2_I_3_/Cs_3_Cu_2_I_5_, and Cs_3_Cu_2_I_5_ with different amounts of OLA-I added, from 0 to 5 mmol. (**c**) Corresponding Tauc plots used for the bandgap estimation of CsCu_2_I_3_ and Cs_3_Cu_2_I_5_. (**d**) PL and PLE spectra of CsCu_2_I_3_, CsCu_2_I_3_/Cs_3_Cu_2_I_5_, and Cs_3_Cu_2_I_5_ with addition of OLA-I from 0 to 5 mmol. (**e**) Time-resolved PL decay curves at room temperature for CsCu_2_I_3_/Cs_3_Cu_2_I_5_ (adding 3 mmol OLA-I) compounds, where solid lines represent the fitting curves obtained via a double exponential function. (**f**) Schematic of the splitting of Cu 3d orbital energy levels in the regular tetrahedral field of CsCu_2_I_3_ and Cs_3_Cu_2_I_5_ ([Cu_2_I_3_]^−^ tetrahedron and [Cu_2_I_5_]^3−^ tetrahedron) according to Jahn–Teller distortion (above), and schematic configuration coordinate diagrams of the luminescence mechanism for CsCu_2_I_3_ and Cs_3_Cu_2_I_5_ (down), respectively (GS1 and GS2 represent the ground states of CsCu_2_I_3_ and Cs_3_Cu_2_I_5_; FE1 and FE2 represent the excited states; STE1 and STE2 are attributed to two emissive self-trapped excitons; PL1 and PL2 denote the photoluminescence of yellow and blue emission).

The optical bandgap and luminescence properties of CsCu_2_I_3_, CsCu_2_I_3_/Cs_3_Cu_2_I_5_, and Cs_3_Cu_2_I_5_ were further explored. As shown in Figure 4a, bright emission was seen under UV illumination, and significant luminescence changes with the different OLA-I treatments occurred from yellow to white, followed by the eventual achievement of blue emission. As shown in Figure 4b, broadband absorption was detected for copper halides, which showed the strongest absorption at around 300 and 330 nm. The bandgaps of the as-prepared CsCu_2_I_3_ and Cs_3_Cu_2_I_5_ were calculated with the Tauc equation, and values of 2.69 eV and 3.61 eV were obtained for Cs_3_Cu_2_I_5_ and CsCu_2_I_3_, respectively (as shown in Figure 4c). The photoluminescence (PL) and photoluminescence excitation (PLE) spectra were obtained, as shown in Figure 4d. CsCu_2_I_3_ exhibited bright yellow emission with the central wavelength at 580 nm and the wavelength of PLE located at 330 nm for 0 mmol OLA-I added. The Cs_3_Cu_2_I_5_ obtained with 5 mmol OLA-I treatment exhibited bright blue emission with a PL maximum centered at 440 nm and maximum excitation peak at around 300 nm. Notably, with OLA-I increased from 0 to 2 mmol, we observed a slight blue shift from 580 to 560 nm and then a gradual red shift to 580 nm, which can be attributed to the quantum confinement effect [66]. Efficient white luminescence characteristics could be realized with OLA-I treatment, which is of great importance for their straightforward and practical WLED application. These compounds can avoid the complex technological processes involved in regulating multiple components in other lead-free metal halides for WLED application [67,68]. 

In Figure S10, the PLE spectra of the CsCu_2_I_3_/Cs_3_Cu_2_I_5_ compounds at different emission wavelengths and the PL spectra of the CsCu_2_I_3_/Cs_3_Cu_2_I_5_ compounds at different excitation wavelengths are presented. Obviously, for the 3 and 4 mmol samples, a mixture of two compounds was obtained and the stable mixed emission of white light was achieved with a certain proportion of OLA-I. The PLE of 300 nm could not only lead to PL at 450 nm but also to weak yellow emission at approximately 580 nm. However, when the wavelength of PLE exceeded 320 nm, only the yellow emission was generated. It can be concluded that when the excitation wavelength increases from 280 to 300 nm, it is beneficial for blue-emissive Cs_3_Cu_2_I_5_, and when the wavelength increases from 310 to 340 nm, it is beneficial for yellow-emissive CsCu_2_I_3_. In summary, with different amounts of OLA-I added during the first dissolution step, CsCu_2_I_3_ undergoes a transformation into a more stabilized Cs_3_Cu_2_I_5_ phase. The time-resolved PL decay curves of the CsCu_2_I_3_/Cs_3_Cu_2_I_5_ compounds were analyzed and their average lifetimes were much longer than those of most perovskites. Figure 4e shows the time-resolved PL spectrum of the CsCu_2_I_3_/Cs_3_Cu_2_I_5_ components, which was measured at two spots and could be well fitted according to the biexponential function. For CsCu_2_I_3_ (emission peak at 580 nm), it presented two decay lifetimes of 1.34 µs and 11.16 µs, with a PL average lifetime τ_ave_ = 5.86 µs, which matched well with a previous study. The obtained lifetime of τ_1_ was 46.03% shorter, and the lifetime of τ_2_ was calculated to be 53.97% longer. Moreover, the average PL lifetime determined for Cs_3_Cu_2_I_5_ (emission peak at 440 nm) at a 300 nm excitation wavelength was measured to be 4.39 µs, and the calculated lifetime of τ_1_ was 53.31% shorter, while the lifetime of τ_2_ was calculated to be 46.69% longer. In addition, the average decay time with 330 nm excitation was 4.86 µs, at which the lifetime of τ_1_ was 52.69% shorter, and the lifetime of τ_2_ was calculated to be 47.31% longer. Based on the above-mentioned results, we attributed the long-lifetime component to the bulk STE emission and the short-lifetime component to the surface trap states, given that STE emission typically exhibits a long lifetime ranging from hundreds of nanoseconds to microseconds. In addition, the time-resolved PL decay curves for other samples are provided in Figures S7–S9 in the Supplementary Materials. All of the samples had a long fluorescence lifetime of microseconds. 

Some recent reports have shown theoretical and experimental results that confirm that the emissions of CsCu_2_I_3_ and Cs_3_Cu_2_I_5_ originate from the formation of self-trapped excitons (STEs) [69,70]. Typically, Jahn–Teller distortion or strong exciton–phonon coupling effects and excited-state structural reorganization are considered to support the PL mechanism. Figure 4f (above) shows the splitting of the Cu 3d orbital energy levels in the regular tetrahedral field of Cu(I) halides; a d^10^ closed shell is beneficial for the tetrahedral geometry. After excitation with a high-energy light, the bandgap photon energy is absorbed, the electronic configuration of Cu(I) 3d^10^ changes to Cu(II) 3d^9^, and the electron moves from the ground state to the excited state and then undergoes intersystem crossing from a singlet to a triplet state (self-trapped state). Originating from the excited-state structural reorganization, Jahn–Teller distortion and the subsequent reorganization of the excited-state structure could subsequently occur. As a result, the energy span varying from the excited state to the self-trapped exciton state makes a major contribution to the length of the Stokes shift. Thus, the excitation and recombination processes for the CsCu_2_I_3_ and Cs_3_Cu_2_I_5_ compounds can be described using the following schematic coordinate diagram provided in Figure 4f (down). In addition, the combination of both blue emission from STE2 in Cs_3_Cu_2_I_5_ and yellow emission from STE1 in CsCu_2_I_3_ can generate efficient white emission. This is different from the widely reported strategies for white-light luminescence. Taking advantage of the luminescence characteristics of CsCu_2_I_3_ and Cs_3_Cu_2_I_5_ is more promising for practical WLED or other applications. 

**Figure 5:**
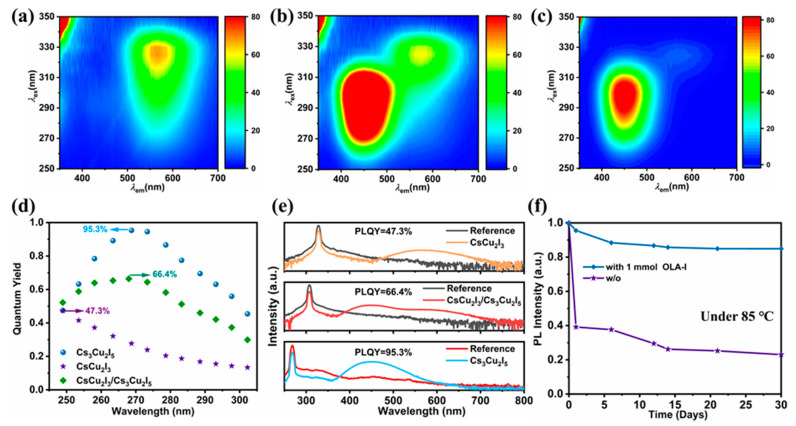
(**a**–**c**) Excitation emission matrix plots of the synthesized CsCu_2_I_3_, CsCu_2_I_3_/Cs_3_Cu_2_I_5_, and Cs_3_Cu_2_I_5_. (**d**) PLQYs of Cs_3_Cu_2_I_5_, CsCu_2_I_3_, and CsCu_2_I_3_/Cs_3_Cu_2_I_5_ with different excitation wavelengths. (**e**) PLQY spectra of the optimal values. (**f**) Evolution of the PL intensity of 0 and 1 mmol OLA-I-treated CsCu_2_I_3_ under 85 °C environment over 30 days for stability investigation.

The dependence of the emission properties on the excitation source is crucial to the performance of copper-based halides in WLED applications. To clearly understand the intrinsic photophysical process, PL excitation–emission maps were obtained, as shown in Figure 5a–c. Clearly, under varied emission and excitation wavelengths, the excitation and emission spectra display almost the same spectral shape and peak position. The emission center wavelength of the synthesized Cs_3_Cu_2_I_5_ is always centered at around 440 nm with the entire range of excitation wavelengths, and that of CsCu_2_I_3_ is centered at around 560 nm. This indicates that our CsCu_2_I_3_ and Cs_3_Cu_2_I_5_ are single-phase, and the broadband emission bands are ascribed to the self-trapped excitons associated with the host lattice. Moreover, for the CsCu_2_I_3_/Cs_3_Cu_2_I_5_ system, two luminescence centers can be identified, which are consistent with CsCu_2_I_3_ and Cs_3_Cu_2_I_5_, respectively. The optical excitation band also has an overlapping component, which is important for the direct fabrication of WLED devices. On the basis of the above discussion, the luminescence property was further investigated by measuring the PL quantum yield (QY), which was recorded via the use of an integration sphere. As seen in Figure 5d, the PLQY of Cs_3_Cu_2_I_5_ is as high as 95.3%, CsCu_2_I_3_ can achieve a value of 47.3%, and CsCu_2_I_3_/Cs_3_Cu_2_I_5_ exhibits a PLQY of 66.4%. All of the PLQY values are listed in Figure 5e, and the PLQYs are comparable to those of samples prepared by other approaches (Table S2). Additionally, the thermostability of CsCu_2_I_3_ compounds was inspected by measuring their PL degradation at the temperature of 85 °C for a long time. In Figure 5f and Figure S11, the evolution of the PL spectra of CsCu_2_I_3_ treated with 0 and 1 mmol OLA-I over 30 days is clearly presented. Obviously, without the addition of OLA-I, the PL intensity decreased tremendously; however, only a slight change was found with 1 mmol OLA-I treatment, even though it was deposited for 30 days. To confirm the stability of the CsCu_2_I_3_/Cs_3_Cu_2_I_5_ component, the sample treated with 3 mmol OLA-I was exposed to air for 60 days. As shown in Figure S12, the PL intensity exhibited no apparent degradation due to the influence of OLA-I, even after storage under ambient conditions for two months. The compound with OLA-I added could retain more than 75% of its initial PL intensity at room temperature and its crystal structure remained unchanged with the passage of time (Figure S13). We also conducted a thermogravimetric analysis (TGA) to demonstrate the improvement in stability through the addition of OLA-I. As illustrated in Figure S14, both the CsCu_2_I_3_ and CsCu_2_I_3_/Cs_3_Cu_2_I_5_ components were measured, and the melting point of CsCu_2_I_3_ was determined at 874 K, which was much lower than that of the CsCu_2_I_3_/Cs_3_Cu_2_I_5_ component. The TGA results of CsCu_2_I_3_/Cs_3_Cu_2_I_5_ demonstrated negligible weight loss under 913 K, suggesting the high thermal stability of CsCu_2_I_3_/Cs_3_Cu_2_I_5_. The inherent stability of the materials determines their large-scale manufacturing capability and facilitates long lifetimes for widespread WLED commercialization. 

**Figure 6:**
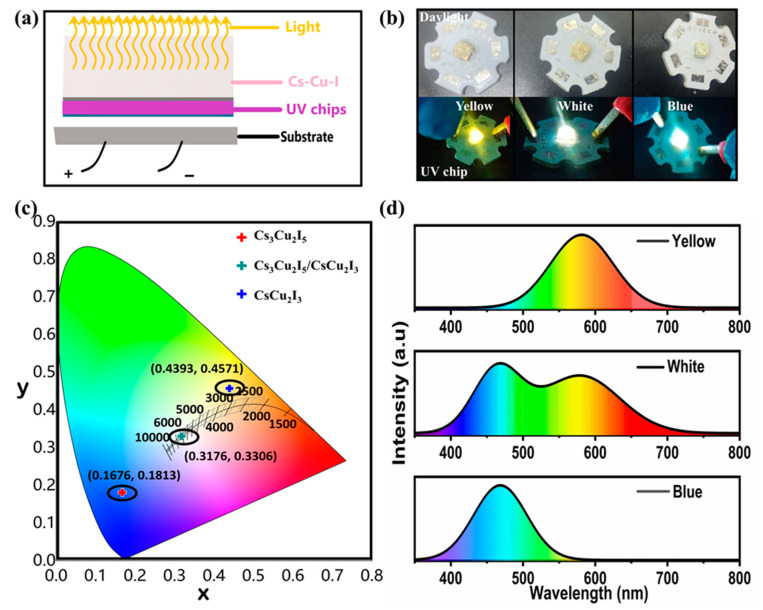
(**a**) Schematic configuration of LED devices fabricated from violet InGaN chips encapsulated with a mixture of Cs-Cu-I samples. (**b**) Photograph of the UV-pumped LED based on CsCu_2_I_3_, CsCu_2_I_3_/Cs_3_Cu_2_I_5_, and Cs_3_Cu_2_I_5_, all operated at a forward bias current of 20 mA (the upper images indicate the LEDs without applying a voltage under daylight). (**c**) CIE chromaticity diagram of the fabricated LED devices based on CsCu_2_I_3_, CsCu_2_I_3_/Cs_3_Cu_2_I_5_, and Cs_3_Cu_2_I_5_, excited by UV light chip (310 nm). (**d**) PL spectra of the working yellow, white, and blue LED devices.

To realize device application, as illustrated in Figure 6a, the as-grown Cs-Cu-I products dispersed in polystyrene (PS) were coated on a commercial UV LED chip (around 310 nm). The corresponding emission photographs can be found in Figure 6b, which demonstrate strong yellow, white, and blue luminescence features when operated at a forward bias current of 20 mA. The calculated Commission Internationale d’Eclairage (CIE) color coordinates of the luminescence of the LED devices are described in Figure 6c. The calculated CIE coordinates were (0.4393, 0.4571) for the yellow LED and (0.1676, 0.1813) for the blue LED. For the white LED, the calculated CIE coordinates, color rendering index (CRI), and correlated color temperature (CCT) values were (0.3176, 0.3306), 90, and 6234 K, which are comparable to those of the light-emitting diodes of other copper-based halides. Additionally, the PL spectra of working yellow, white, and blue LED devices are further presented in Figure 6d, which are consistent with the system of Cs-Cu-I powder samples. These results suggest the great promise of these copper halides for practical LED application. Specifically, we summarize the recent advances in the preparation of a variety of unique copper halides and several representative works are shown in Table S2 [46,53,71,72,73,74,75,76,77,78,79]. Considering the existing progress achieved in multifarious optoelectronic applications and their associated challenges, the further development described in this work provides a new perspective for the engineering of the luminance properties of copper halides through modification with OLA-I treatment.

## 3. Materials and Methods

Chemicals. The chemicals included the following: copper(I) iodide (CuI, Sigma (St. Louis, MO, USA), 99.9%), copper(I) chloride (CuCl, Sigma (St. Louis, MO, USA), 99.9%), cesium carbonate (Cs_2_CO_3_, Aladdin (Shanghai, China), 99.9%), 1-octadecene (ODE, Macklin (Shanghai, China), 90%), oleic acid (OA, Aladdin (Shanghai, China), 85%), oleylammonium (OLA, Adamas-beta (Shanghai, China), 90%+), hydroiodic acid (HI, Aladdin, 58 wt%), and isopropyl alcohol (IPA, Sigma-Aldrich, (St. Louis, MO, USA) ≥99.7%). All chemicals were used as purchased, without further purification.

Synthesis of Cs Oleate (0.4 M). Cs_2_CO_3_ (3.26 g), oleic acid (OA, 15.8 mL), and 1-octadecene (ODE, 34.2 mL) were added to a 100 mL three-neck flask, dried at 120 °C for 1 h, and then heated under nitrogen to 150 °C, which was maintained for a period of time. A clear and transparent solution could be obtained until all Cs_2_CO_3_ had reacted with the oleic acid, and then the product was kept in a glass bottle for subsequent use. It should be noted that the Cs oleate precipitated from ODE at room temperature had to be preheated to 100 °C before further injection.

Synthesis of OLA-I (2.2 M). HI (12 mL) and OLA (30 mL) were added to a 100 mL three-neck flask, and the reaction temperature was elevated gradually to avoid a violent reaction. It was then dried at approximately 100 °C under a nitrogen/vacuum environment for another 1 h to guarantee complete reaction. The product was kept in a glass bottle and was preheated to 100 °C to achieve a homogeneous solution before further steps.

Synthesis of CsCu_2_I_3_ MRs. CuI (762 mg, 4 mmol), OA (8 mL), OLA (8 mL), and ODE (40 mL) were added to a 100 mL three-neck flask and dried under a vacuum environment at 120 °C for 40 min. Subsequently, the temperature was increased to 150 °C to ensure that the CuI salt dissolved completely and formed a transparent solution with oleic acid and oleylamine under a N_2_ atmosphere. Then, the reaction temperature was kept at 150 °C and the previously synthesized Cs oleate precursor (4 mL) was swiftly injected into the aforementioned solution. After a 30 s reaction under continuous magnetic agitation, the solution containing the CsCu_2_I_3_ MRs was quickly cooled to room temperature using an ice-water bath. The crude solution was first separated through high-speed centrifugation at 8000 rpm for 5 min. The supernatant was discarded after centrifugation, and the precipitate was redispersed in isopropanol and centrifuged at 6000 rpm for 5 min again. Finally, the precipitate was collected in the centrifuge tube and hermetically preserved for characterization and application.

Synthesis of CsCu_2_I_3_/Cs_3_Cu_2_I_5_ composites and Cs_3_Cu_2_I_5_ NCs. Different amounts of OLA-I (1–5 mmol) were introduced into the 100 mL three-necked flask in the first procedure during the synthesis of CsCu_2_I_3_ MRs, while other synthetic parameters were kept the same.

Fabrication of WLED devices. Firstly, polystyrene (PS, 1.0 g) was added to 3 mL of toluene and stirred at room temperature until a transparent PS/toluene solution (25 wt%) was obtained. Afterwards, the air-dried composite powders (CsCu_2_I_3_/Cs_3_Cu_2_I_5_) were thoroughly mixed in the above-mentioned solution and the mixture was spread on the surface of an ultraviolet LED chip with a wavelength of 310 nm.

### Characterization

The crystal structures of the as-prepared samples were measured using an X-ray diffractometer (XRD, Rikagu Ultima III, Tokyo, Japan) equipped with monochromatized Cu Kα radiation (λ = 1.5418 Å). The morphologies of the products were examined using scanning electron microscopy (SEM) and energy-dispersive X-ray spectroscopy (EDS), which were performed on a JEOL JSM-7800F (Tokyo, Japan) device at 10 kV. X-ray photoelectron spectroscopy (XPS) measurements were recorded using an achromatic Al Kα source (1486.6 eV) and a double-pass cylindrical mirror analyzer (ULVAC-PHI 5000 Versa Probe, Tokyo, Japan). Transmission electron microscopy (TEM) measurements were executed using a FEI Tecnai G2 F20 electron microscope (FEI Company, Hillsboro, OR, USA) under 200 kV. TG data were collected with a NETZSCH thermalgravimetric analyzer (German Netzche Company, Selb, Germany) and the testing range was from room temperature to 1000 °C. Ultraviolet and visible absorption (UV–vis) spectra were recorded using a Shimadzu UV-3600 plus spectrophotometer (Tokyo, Japan) under room temperature. Photoluminescence excitation (PLE) and photoluminescence (PL) spectra were recorded using a Horiba PTI Quanta Master 400 fluorescence spectrometer (Tokyo, Japan) under ambient conditions. The absolute photoluminescence quantum yields (PLQYs) were measured using a Horiba PTI Quanta Master 400 steady-state fluorescence system with an integrated sphere and double-checked with a Hamamatsu Photonics Quantaurus-QY device (model: C11347-11). The fluorescence decay processes were recorded under ambient conditions with the time-correlated single-photon counting (TCSPC) technique, on a Nikon Ni-U Microfluorescence Lifetime System (Confotec MR200, SOL, Minsk, Belarus) with a 330 nm picosecond laser.

## 4. Conclusions

In summary, we have demonstrated a controllable strategy for the preparation of copper iodide nanocrystals through alkylammonium halide modification. Bright yellow-emission CsCu_2_I_3_, blue-emission Cs_3_Cu_2_I_5_, and white-emission CsCu_2_I_3_/Cs_3_Cu_2_I_5_ can be achieved by controlling the ratio of OLA-I during synthesis. The emission of the 1D CsCu_2_I_3_ and 0D Cs_3_Cu_2_I_5_ originates from self-trapped excitons, which is demonstrated by their broad emission spectra with large Stokes shifts and microsecond decay times. The yellow PL peak for CsCu_2_I_3_ is centered at 560 nm with a PLQY of 47.3%, the blue PL peak for Cs_3_Cu_2_I_5_ is located at 440 nm with an unprecedentedly high PLQY of 95.3%, and the white emission also exhibits a PLQY of 66.4%. They all show great application potential for LEDs. Consequently, a high-quality and stable WLED was fabricated by blending the as-prepared copper halides and polystyrene glue with commercial UV LED chips. For the WLED, the calculated CIE coordinates, color rendering index (CRI), and correlated color temperature (CCT) values were (0.3176, 0.3306), 90, and 6234 K, respectively. Together with their eco-friendly features, low-cost processability, and nontoxic nature, these copper halide nanocrystals can therefore be regarded as attractive alternative and reliable light emitters for high-performance LEDs. While lead-free cesium copper halide nanocrystals are in high demand due to their unprecedentedly high emission intensities, this approach, leading to high quantum yields, would certainly contribute to future research in the LED field.

## Data Availability

Data are contained within the article and Supplementary Materials.

## Supplementary Materials

The following supporting information can be downloaded at: https://www.mdpi.com/article/10.3390/molecules29051162/s1, Figure S1. Energy-dispersive spectra (EDS) and corresponding elemental content of compounds obtained with different molar ratios of OLA-I added: (a–f) 0 mmol; 1 mmol; 2 mmol; 3 mmol; 4 mmol; and 5 mmol. Figure S2. XRD patterns of the samples with enlarged views from 25° to 30°. Figure S3. Transmission electron microscope (TEM) images of CsCu_2_I_3_ MRs: (a) low resolution and (b) high resolution; TEM images of Cs_3_Cu_2_I_5_ NCs: (c) low-resolution images and (d) high-resolution images. Figure S4. (a, b) The diameter and length distribution statistics of CsCu_2_I_3_ MRs obtained with 1 mmol OLA-I added. (c) The particle size and diameter distribution statistics of Cs_3_Cu_2_I_5_ NCs. Figure S5. (a) XPS survey spectrum of CsCu_2_I_3_ and high-resolution spectra corresponding to the curves of (b) Cs 3d, (c) Cu 2p, and (d) I 3d orbitals. Figure S6. (a) XPS survey spectrum of Cs_3_Cu_2_I_5_ and high-resolution XPS spectra of (b) Cs 3d, (c) Cu 2p, and (d) I 3d orbitals. Figure S7. Time-resolved PL decay curves at room temperature for CsCu_2_I_3_ (with addition of 0 mmol, 1 mmol, and 2 mmol OLA-I) compound, where solid lines represent the fitting curves using a double exponential function. Figure S8. Time-resolved PL decay curves at room temperature for CsCu_2_I_3_/Cs_3_Cu_2_I_5_ (with addition of 4 mmol OLA-I) compound, where solid lines represent the fitting curves using a double exponential function. Figure S9. Time-resolved PL decay curves at room temperature for Cs_3_Cu_2_I_5_ (with addition of 5 mmol OLA-I) compound, where solid lines represent the fitting curves using a double exponential function. Figure S10. (a) PLE spectra of CsCu_2_I_3_/Cs_3_Cu_2_I_5_ compound for different emission wavelengths. (b) PL spectra of CsCu_2_I_3_/Cs_3_Cu_2_I_5_ compound upon UV irradiation with different excitation wavelengths. Figure S11. (a) Evolution of the PL spectra of 0 mmol OLA-I-treated CsCu_2_I_3_ under 85°C over 30 days for stability investigation. (b) Evolution of the PL spectra of 1 mmol OLA-I-treated CsCu_2_I_3_ over 30 days for stability investigation (inserted images representing the luminance change under an 85 °C environment: upper—for 0 days; lower—for 30 days). Figure S12. PL emission spectrum of the obtained compound with addition of 3 mmol OLA-I exposed to air for 60 days. Figure S13. XRD patterns of 3 mmol OLA-I-treated CsCu_2_I_3_/Cs_3_Cu_2_I_5_ component before and after storage under ambient conditions for two months. Figure S14. XRD patterns of 3 mmol OLA-I-treated CsCu_2_I_3_/Cs_3_Cu_2_I_5_ component before and after storage under ambient conditions for two months. Table S1. Element analysis measurement results and the calculated ratios of Cs, Cu, and I for prepared samples with addition of different molar ratios of OLA-I (ratio of input amounts for Cs:Cu:I = 3.2:4:4). Table S2. Summary of the synthetic strategies and optical parameters of recently reported copper-based halide compounds.
