# Supplementary material for: Alkylammonium Halides for Phase Regulation and Luminescence Modulation of Cesium Copper Iodide Nanocrystals for Light-Emitting Diodes
Source: Molecules. 2024 Mar 5;29(5):1162. doi: 10.3390/molecules29051162 (PMC10934009; doi:10.3390/molecules29051162)
Supplement: Supplementary file 1 [file molecules-29-01162-s001.zip › molecules-2828466-supplementary.pdf]

Electronic Supplementary Information (ESI)

AAlkylammonium Halides for Phase Regulation and Luminescence Modulation of Cesium Copper Iodide Nanocrystals for Light-Emitting Diodes

Wen Meng, Chuying Wang, Guangyong Xu, Guigen Luo and Zhengtao Deng *

College of Engineering and Applied Sciences, State Key Laboratory of Analytical Chemistry for Life Science, Na-tional Laboratory of Microstructures, Nanjing University, Nanjing 210023, China;

17351930477@163.com (W.M.); wangchuying@yeah.net (C.W.); xuguangyong@smail.nju.edu.cn (G.X.); 502022340061@smail.nju.edu.cn (G.L.)

* Correspondence: dengz@nju.edu.cn

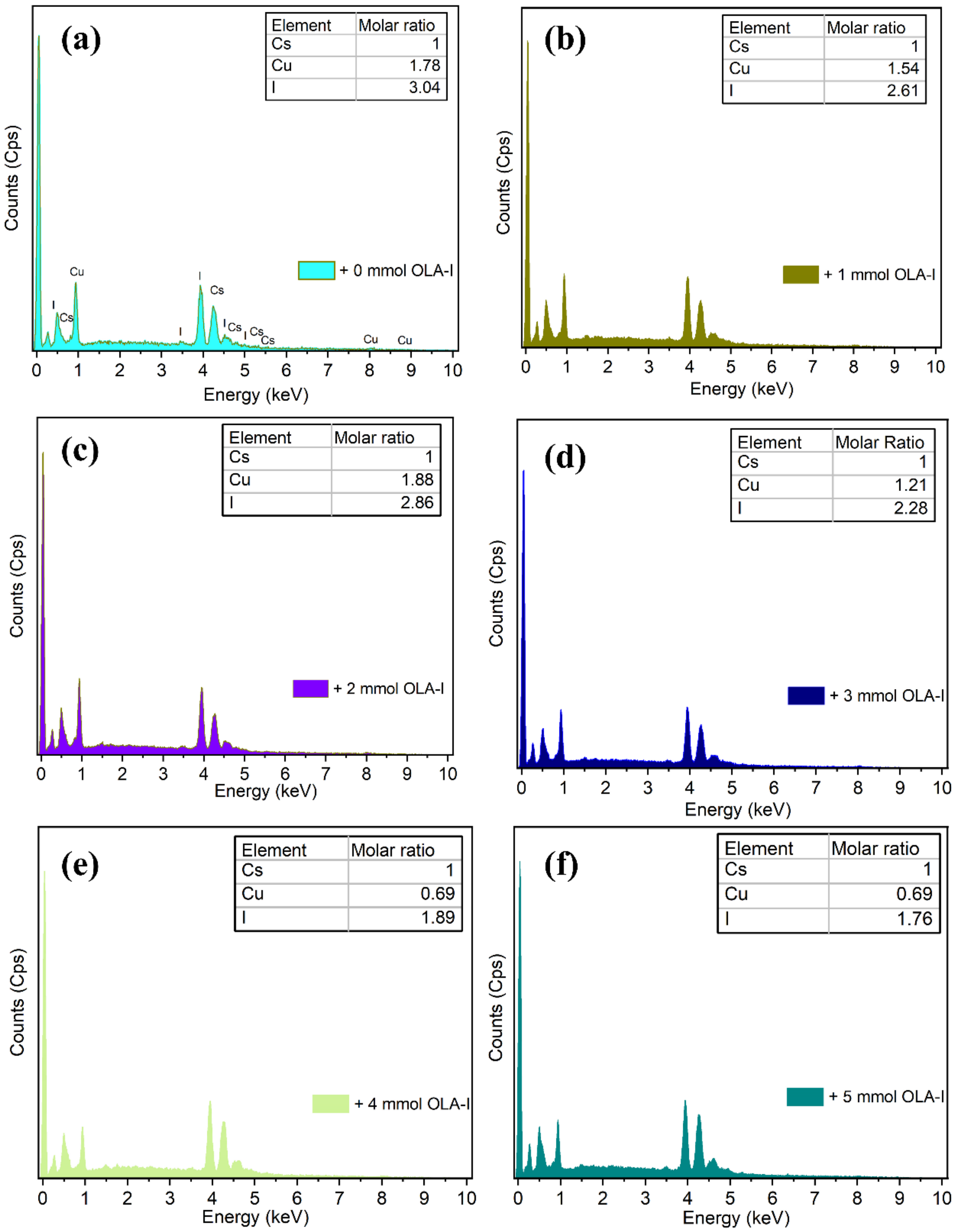

Figure S1. The Energy dispersive spectra (EDS) and corresponding elemental content of compounds obtained with different molar ratio OLA-I added: (a-f) 0 mmol; 1 mmol; 2 mmol; 3 mmol; 4 mmol and 5 mmol OLA-I.

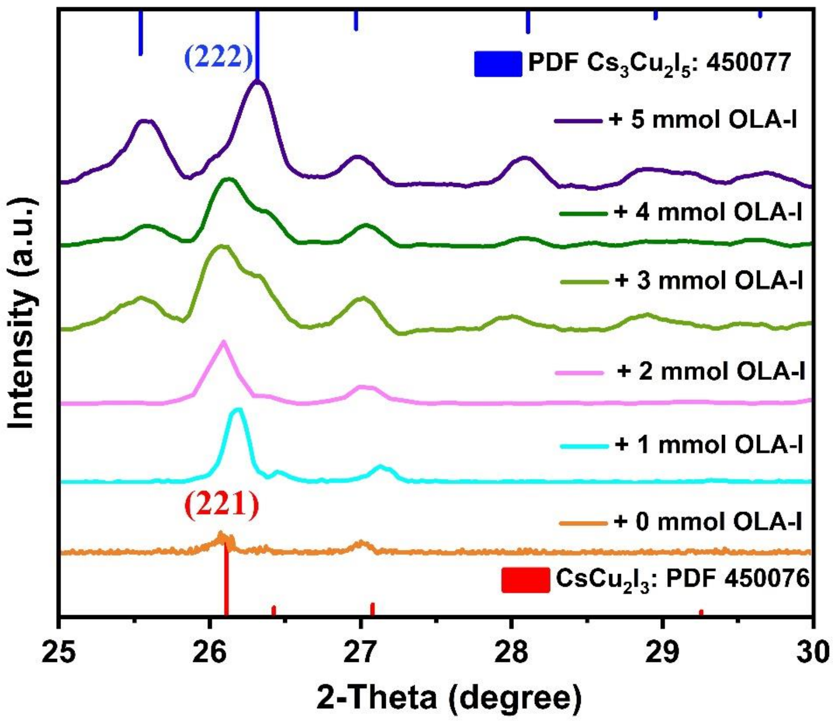

Figure S2. XRD patterns of the samples with enlarged views from 25° to 30°.

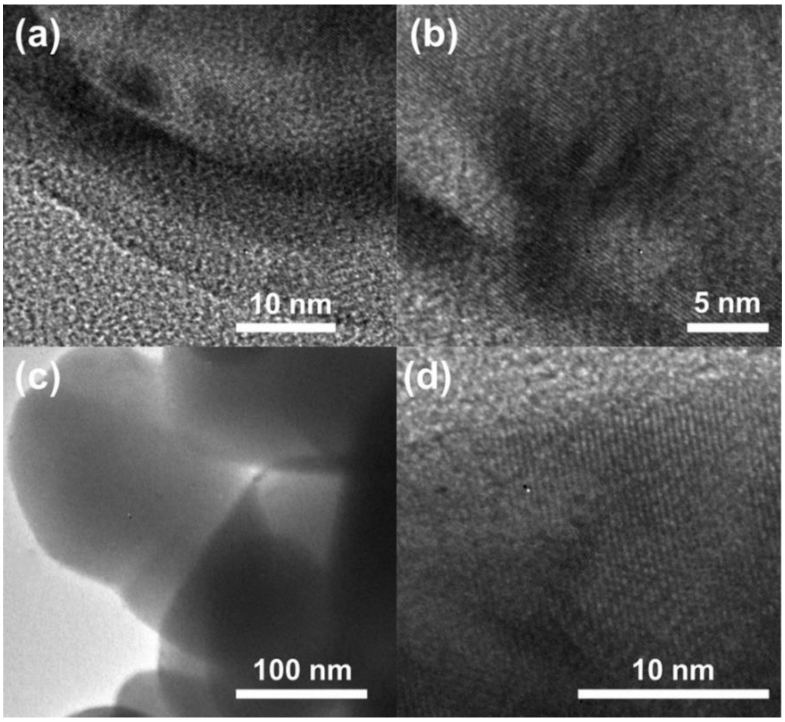

Figure S3. The transmission electron microscope (TEM) images of $CsCu_2I_3$ MRs (a) low-resolution and (b) high-resolution; TEM images of $Cs_3Cu_2I_5$ NCs (c) low-resolution image and (d) high-resolution image.

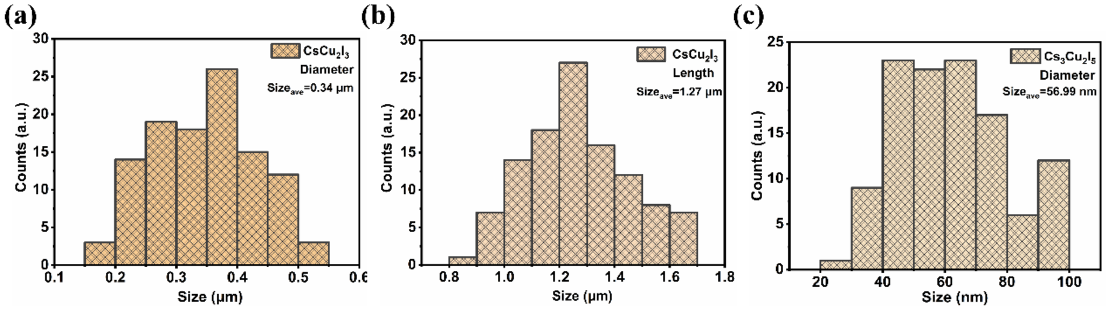

Figure S4. (a-b) The diameter and length distribution statistics of $CsCu_2I_3$ MRs obtained through 1 mmol OLA-I added. (c) The particles size of diameter distribution statistic of $Cs_3Cu_2I_5$ NCs.

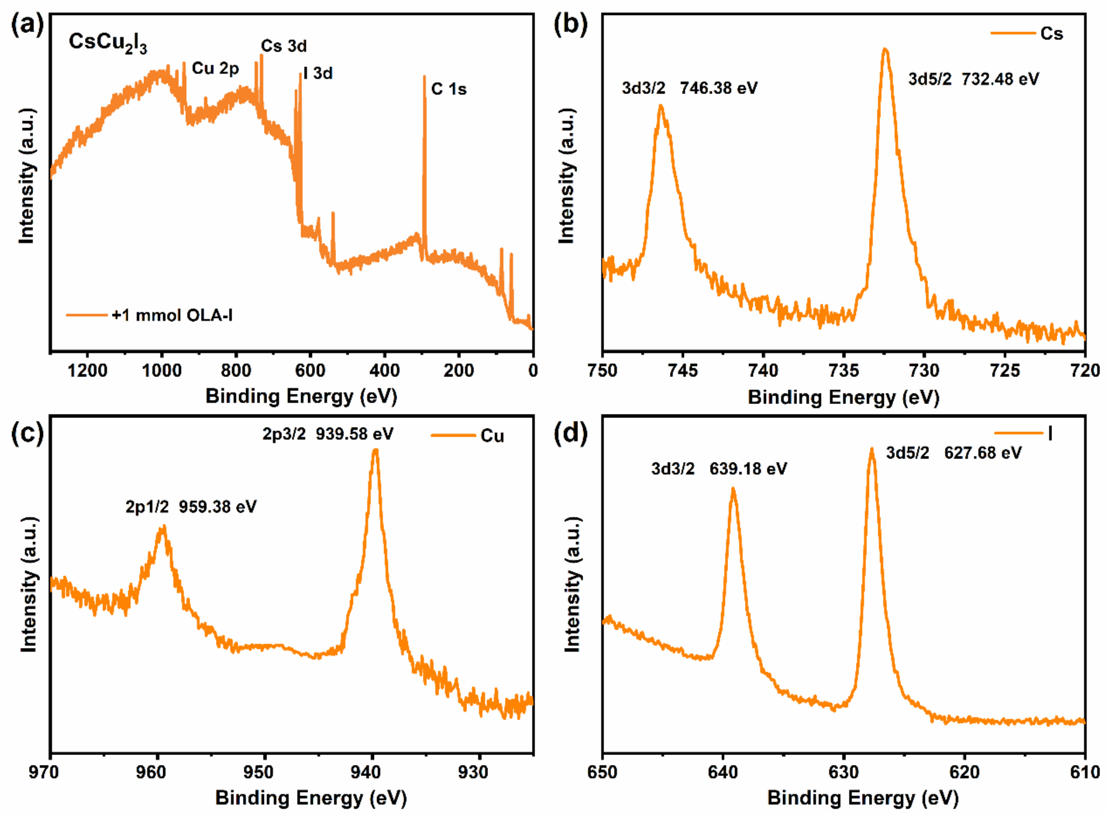

Figure S5. (a) XPS survey spectrum of $CsCu_2I_3$. And the high-resolution spectra corresponding to the curves of (b) Cs 3d, (c) Cu 2p and (d) I 3d orbitals.

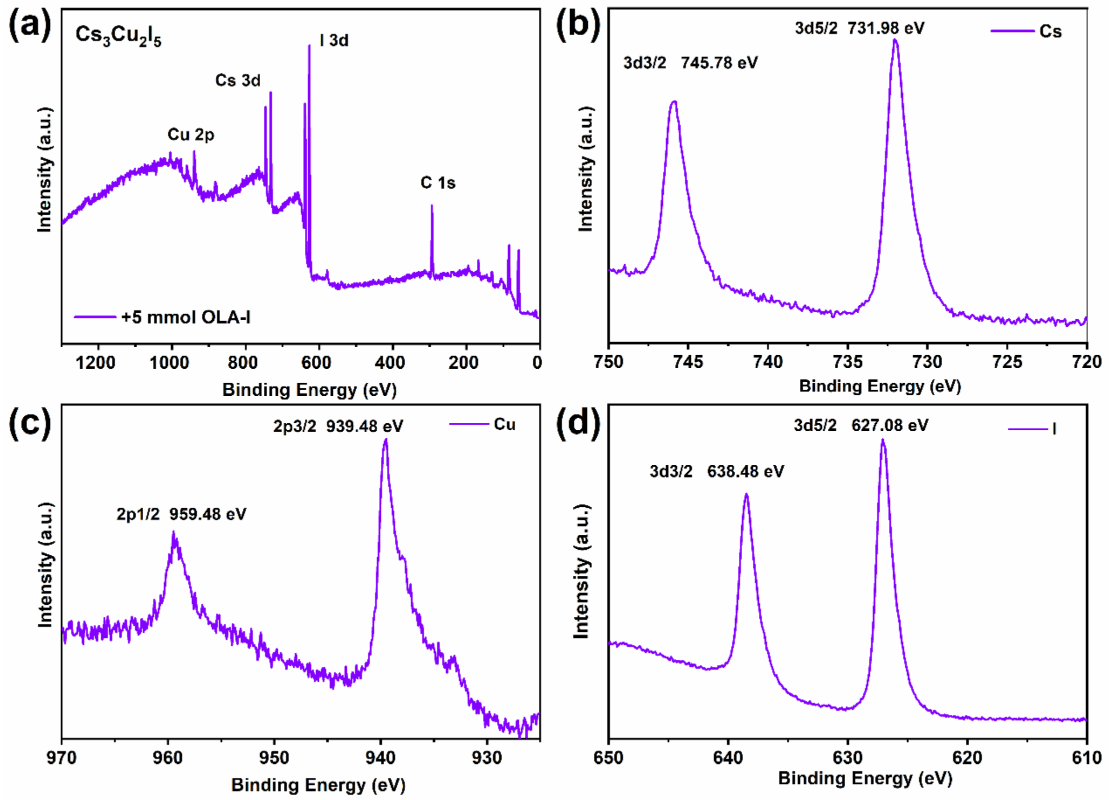

Figure S6. (a) XPS survey spectrum of $Cs_3Cu_2I_5$. And the high-resolution XPS spectra of (b) Cs 3d, (c) Cu 2p and (d) I 3d orbitals.

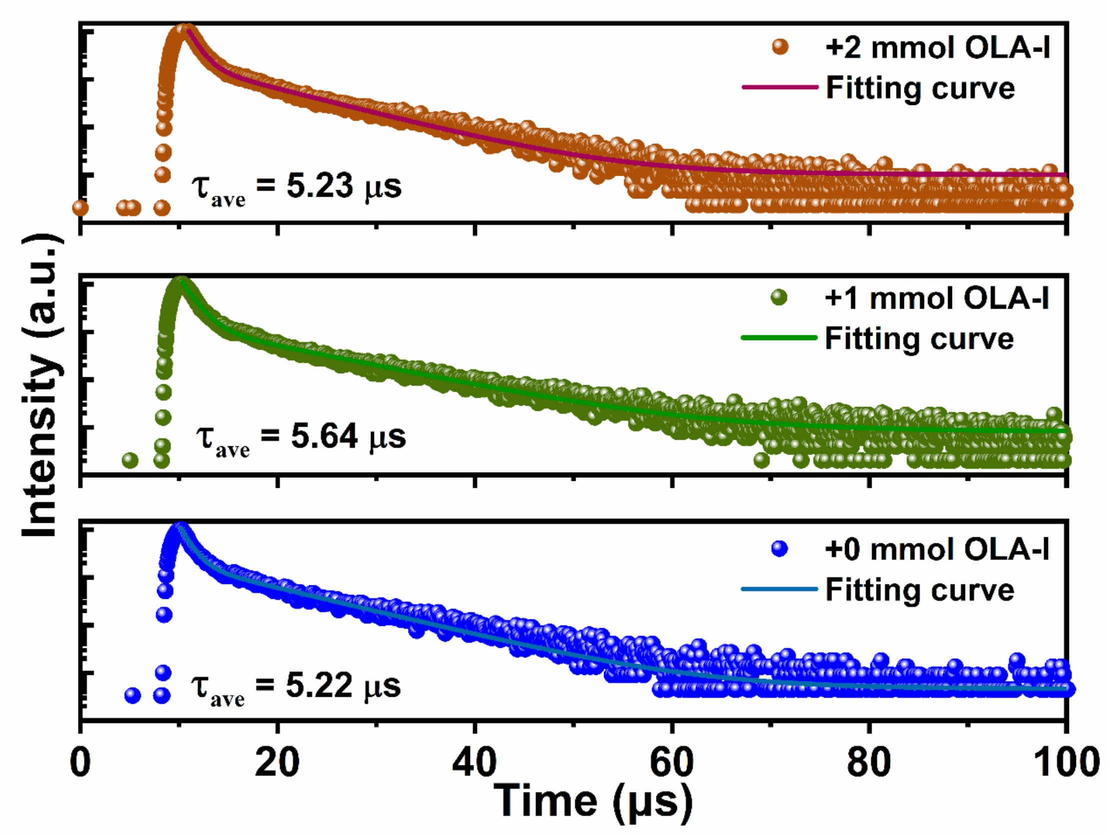

Figure S7. Time-resolved PL decay curves at room temperature for $CsCu_2I_3$ (adding 0 mmol, 1 mmol and 2 mmol OLA-I) compound and solid lines represent the fitting curves by a double exponential function.

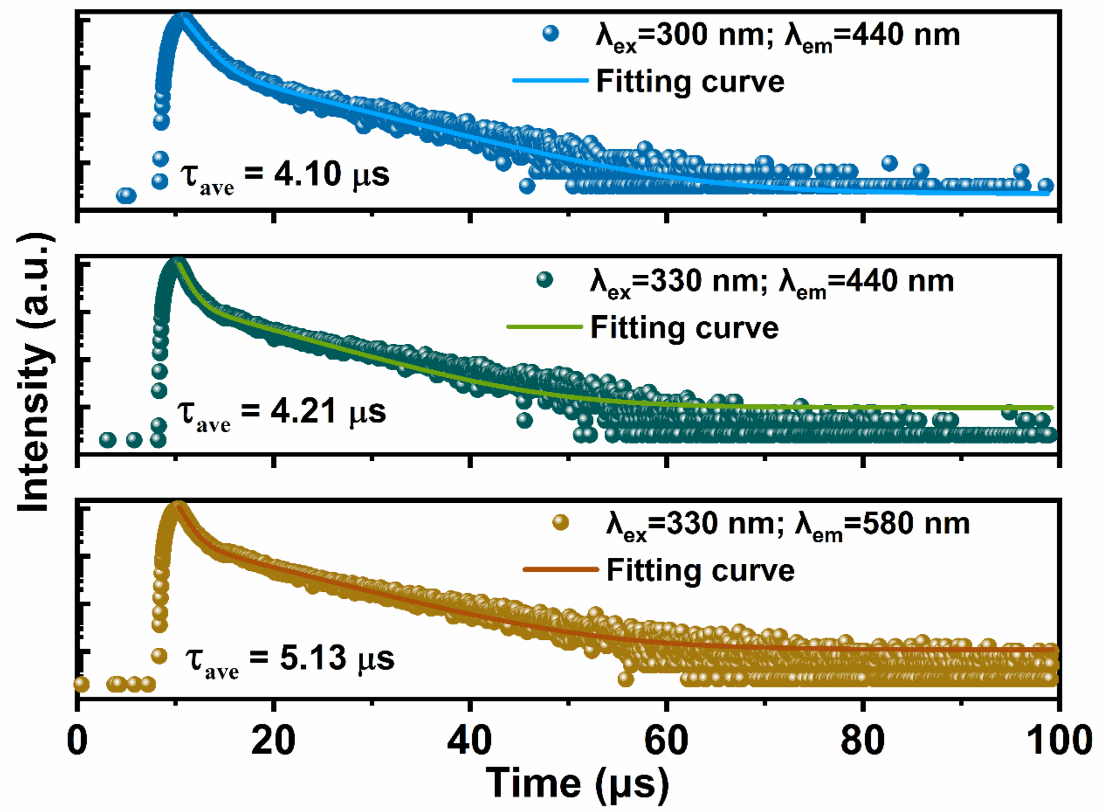

Figure S8. Time-resolved PL decay curves at room temperature for $CsCu_2I_3/Cs_3Cu_2I_5$ (adding 4 mmol OLA-I) compound and solid lines represent the fitting curves by a double exponential function.

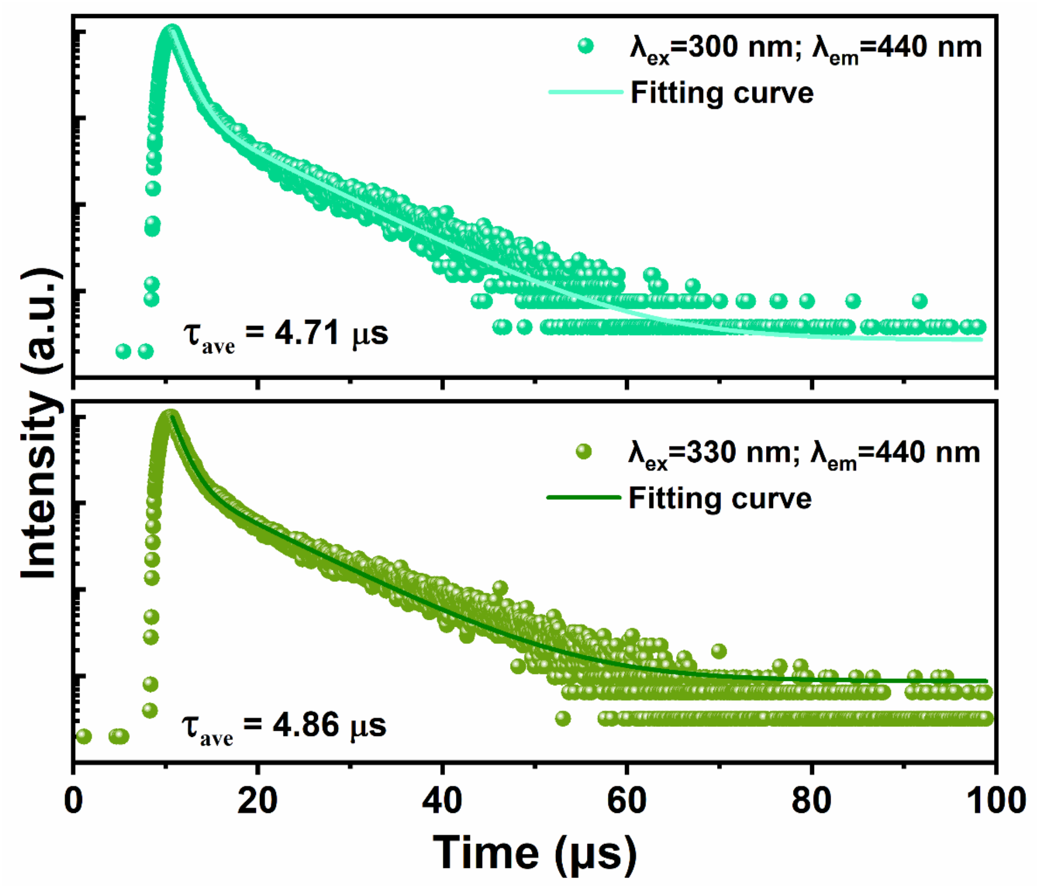

Figure S9. Time-resolved PL decay curves at room temperature for $Cs_3Cu_2I_5$ (adding 5 mmol OLA-I) compound and solid lines represent the fitting curves by a double exponential function.

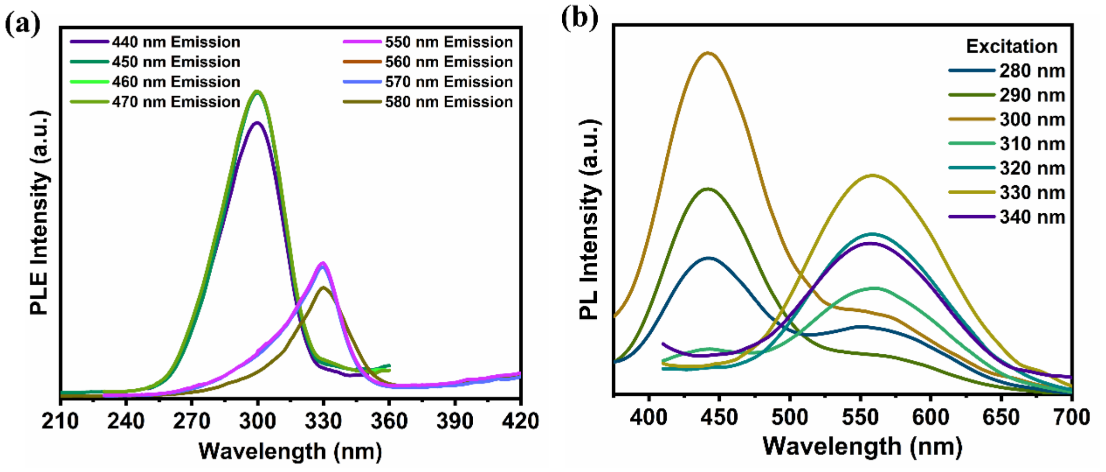

Figure S10. (a) PLE spectra of $CsCu_2I_3/Cs_3Cu_2I_5$ compound for different emission wavelengths. (b) PL spectra of $CsCu_2I_3/Cs_3Cu_2I_5$ compound upon UV irradiation with different excitation wavelengths.

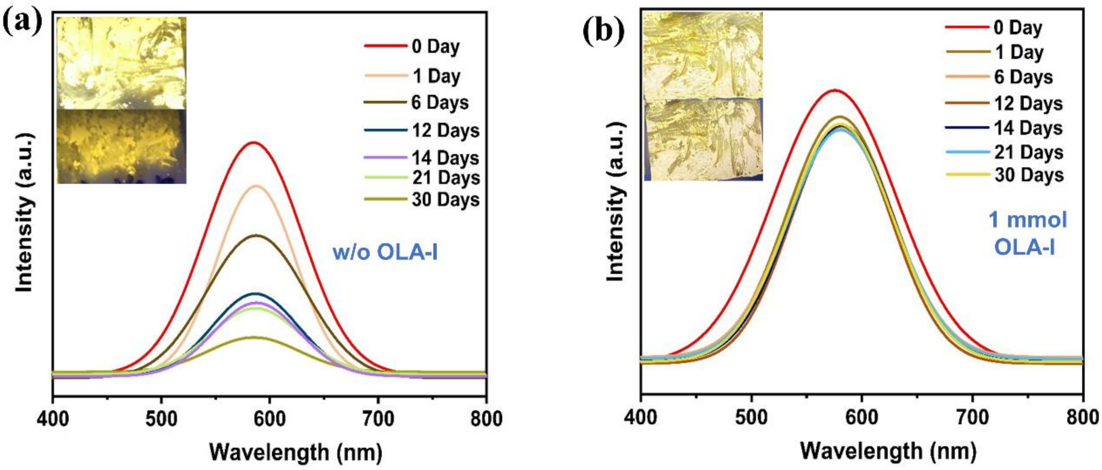

Figure S11. (a) Evolution of the PL spectra of 0 mmol OLA-I treated $CsCu_2I_3$ under 85 ℃ over 30 days for stability investigation. (b) Evolution of the PL spectra of 1 mmol OLA-I treated $CsCu_2I_3$ over 30 days for stability investigation (Inserted images representing the luminance changing under 85 ℃ environment. Above: for 0 day; Down: for 30 days).

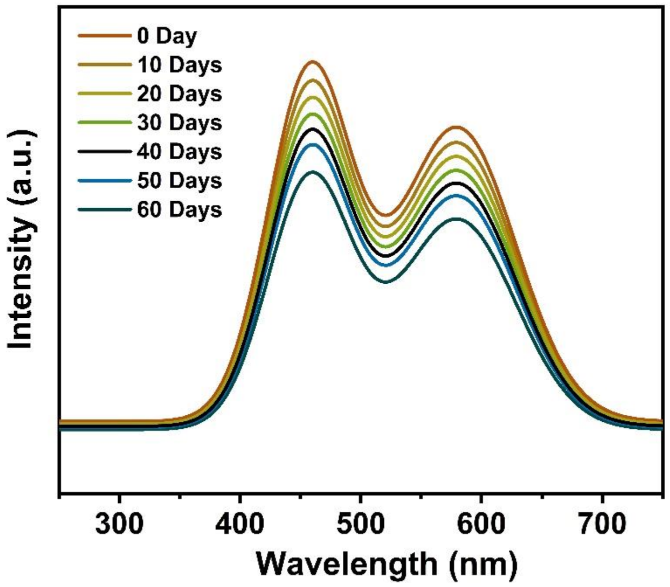

Figure S12. PL emission spectrum of the obtained compound with adding 3 mmol OLA-I exposed to air for 60 days.

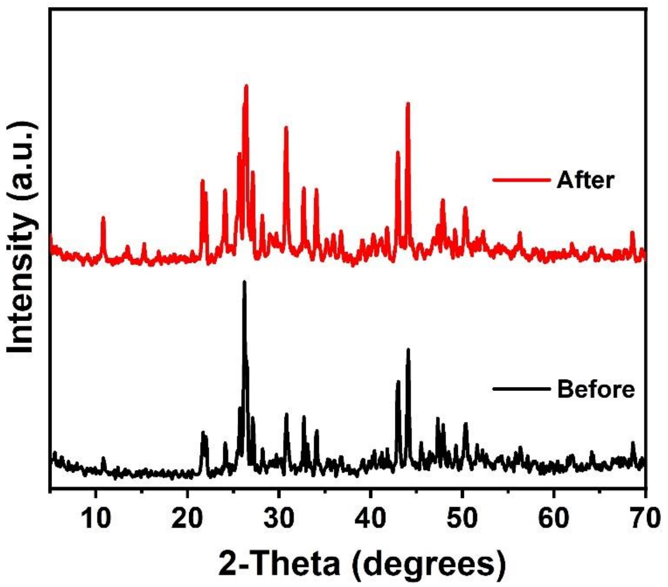

Figure S13. XRD patterns of 3 mmol OLA-I treated $CsCu_2I_3/Cs_3Cu_2I_5$ component before and after storage under the ambient condition for two months.

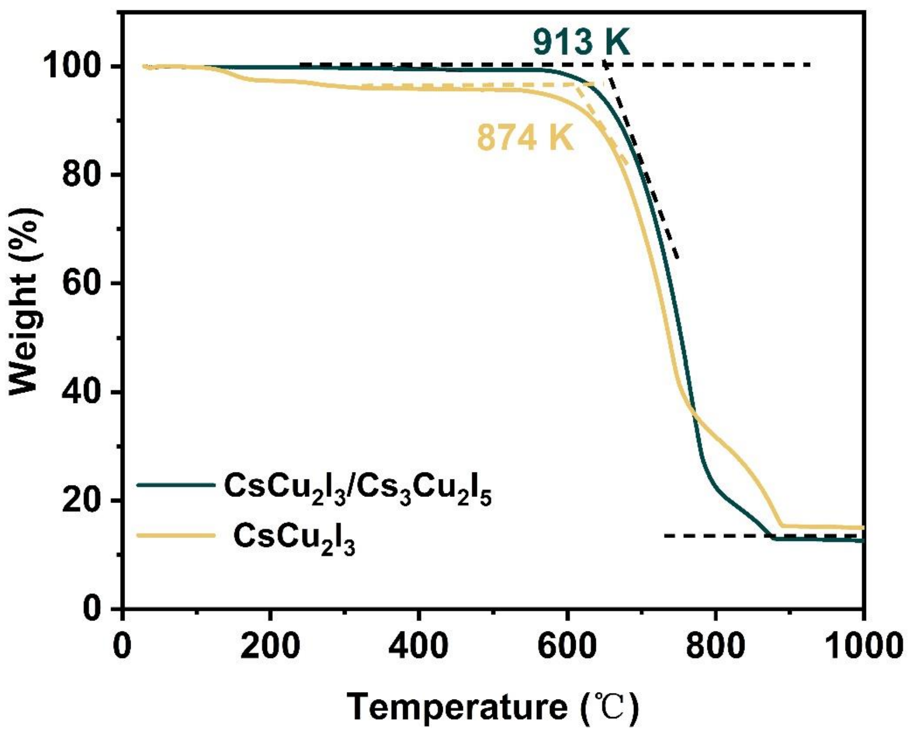

Figure S14. Thermogravimetric analysis (TGA) of $CsCu_2I_3$ and $CsCu_2I_3/Cs_3Cu_2I_5$.

Table S1. Element analysis measurement results and the calculated ratios of Cs, Cu and I for prepared samples with adding different molar ratio of OLA-I (Ratio of inputting amounts for Cs: Cu: I=3.2: 4: 4).

+ x mmol OLA-I	Cs (%)	Cu (%)	I (%)	Ratio	Component
**+ 0 mmol**	13.8	24.6	41.9	1:1.78:3.04	**$CsCu_2I_3$**
**+ 1 mmol**	14.2	21.8	36.9	1:1.54:2.61	**$CsCu_2I_3$**
**+ 2 mmol**	12.5	23.5	35.7	1:1.88:2.86	**$CsCu_2I_3$**
**+ 3 mmol**	15.7	19.0	35.8	1:1.21:2.28	**$CsCu_2I_3$**<br>**$Cs_3Cu_2I_5$**
**+ 4 mmol**	22.1	15.3	41.7	1:0.69:1.89	**$CsCu_2I_3$**<br>**$Cs_3Cu_2I_5$**
**+ 5 mmol**	20.2	13.9	35.6	1:0.69:1.76	**$Cs_3Cu_2I_5$**

Table S2. Summary of the synthetic strategies and optical parameters of recent reported copper-based halide compounds.

Formula	Morphology	Method	Emission peak (nm)	PLQY (%)	Ref.
$CsCu_2I_3$	NA	Solid-state reaction	576	3.23	[1]
$CsCu_2I_3$	Thin films	Spin-coating	~ 548	20.6	[2]
$CsCu_2I_3$	Micro-rods	Antisolvent infiltration	575	12.1	[3]
$CsCu_2I_3$	Nanorods	Hot injection	553	5	[4]
$CsCu_2I_3$	Nanorods	Hot injection	561	11	[5]
$CsCu_2I_3$	Wires	Antisolvent	570	NA	[6]
$Cs_3Cu_2I_5$	Nanocrystals	Hot injection	441	67	[4]
$Cs_3Cu_2I_5$	Nanocrystals	Modified hot injection	445	73.7	[7]
$Cs_3Cu_2I_5$	Powder	Solution stirring	440	NA	[8]
$Cs_3Cu_2I_5$: Mn	Microparticles	Solid-state reaction	448 and 556	57	[9]
$Cs_3Cu_2I_5$	Nanocrystals	Hot injection	441	67	[10]
$Cs_5Cu_3Cl_6I_2$	Powder	Solid-state reaction	462	95	[11]
**$CsCu_2I_3$**	**Micro-rods**	**Hot injection**	**560**	**47.3**	**This work**
**$CsCu_2I_3$/ $Cs_3Cu_2I_5$**	**Micro-rods and Nanocrystals**	**Hot injection**	**560 and 440**	**66.4**	**This work**
**$Cs_3Cu_2I_5$**	**Nanocrystals**	**Hot injection**	**440**	**95.3**	**This work**

Additional References

[1] R. Roccanova, A. Yangui, G. Seo, T. D. Creason, Y. Wu, D. Y. Kim, M.-H. Du, B. Saparov, *ACS Materials Letters* **2019**, *1*, 459.

[2] Z. Ma, Z. Shi, C. Qin, M. Cui, D. Yang, X. Wang, L. Wang, X. Ji, X. Chen, J. Sun, D. Wu, Y. Zhang, X. J. Li, L. Zhang, C. Shan, *ACS Nano* **2020**, *14*, 4475.

[3] S. Fang, Y. Wang, H. Li, F. Fang, K. Jiang, Z. Liu, H. Li, Y. Shi, *Journal of Materials Chemistry C* **2020**, *8*, 4895.

[4] P. Cheng, L. Sun, L. Feng, S. Yang, Y. Yang, D. Zheng, Y. Zhao, Y. Sang, R. Zhang, D. Wei, W. Deng, K. Han, *Angewandte Chemie International Edition* **2019**, *58*, 16087.

[5] P. Vashishtha, G. V. Nutan, B. E. Griffith, Y. Fang, D. Giovanni, M. Jagadeeswararao, T. C. Sum, N. Mathews, S. G. Mhaisalkar, J. V. Hanna, T. White, *Chemistry of Materials* **2019**, *31*, 9003.

[6] X. Xing, T. Tong, M. Mohebinia, D. Wang, Z. Ren, V. G. Hadjiev, Z. Wang, J. Bao, *The Journal of Physical Chemistry Letters* **2022**, *13*, 6447.

[7] L. Lian, M. Zheng, W. Zhang, L. Yin, X. Du, P. Zhang, X. Zhang, J. Gao, D. Zhang, L. Gao, G. Niu, H. Song, R. Chen, X. Lan, J. Tang, J. Zhang, *Advanced Science* **2020**, *7*, 2000195.

[8] X. Zhang, B. Zhou, X. Chen, W. W. Yu, *Inorganic Chemistry* **2022**, *61*, 399.

[9] P. Du, P. Cai, W. Li, L. Luo, Y. Hou, Z. Liu, *Microchimica Acta* **2019**, *186*, 730.

[10] L. Liu, D. Zhou, L. Zi, R. Sun, S. Liu, B. Liu, Z. Shi, D. Liu, H. Song, *Solar RRL* **2022**, *6*, 2200025.

[11] J. Li, T. Inoshita, T. Ying, A. Ooishi, J. Kim, H. Hosono, *Advanced Materials* **2020**, *32*, 2002945.
